# Impact of visceral obesity on postoperative outcomes in colorectal cancer: a systematic review and meta-analysis

**DOI:** 10.3389/fonc.2025.1538073

**Published:** 2025-05-06

**Authors:** Yulong Wang, Xijuan Liu, Xiao Feng, Xing Jiang, Lili Huang

**Affiliations:** ^1^ Department of Gastrointestinal Surgery, Xinghua People’s Hospital Affiliated to Yangzhou University, Xinghua, China; ^2^ School of Public Health, Shandong Second Medical University, Weifang, China; ^3^ Department of Cardiology, Xinghua People’s Hospital Affiliated to Yangzhou University, Xinghua, China

**Keywords:** colorectal cancer, visceral obesity, visceral fat area, surgery, complications

## Abstract

**Background:**

This systematic review and meta-analysis aimed to assess the impact of visceral obesity (VO) on postoperative outcomes in colorectal cancer (CRC) patients.

**Methods:**

Primary studies were obtained from sources like Embase, PubMed, and Web of Science during the search, which ran until October 2024. Patients with colorectal cancer who had visceral obesity (VO) and those who did not were compared in terms of intraoperative conditions, postoperative outcomes, postoperative complications, and long-term prognoses, including overall survival (OS) and disease-free survival (DFS).

**Results:**

5,756 individuals with VO and 5,373 patients without VO were among the 11,129 patients who had colorectal cancer resected. Patients with VO had higher conversion rates (p = 0.03), fewer lymph nodes removed (p = 0.05), and longer recovery times for bowel movements (p = 0.009). Furthermore, patients with VO had a considerably greater overall incidence of sequelae than those without (p = 0.0003), including anastomotic leaks (p = 0.01), intestinal obstruction (p = 0.0003), intra-abdominal abscesses (p = 0.004), wound infections (p < 0.00001), and pulmonary problems (p = 0.0003). OS and DFS, however, did not differ between the two groups (p > 0.05).

**Conclusions:**

Colorectal cancer patients with VO who have surgery tend to have fewer lymph nodes taken, more problems after surgery, and a higher rate of switching to open surgery.

## 1 Introduction

Colorectal cancer (CRC) is one of the most common gastrointestinal malignancies globally, ranking third in incidence and second in mortality (1). Although curative surgery remains the most effective treatment for colorectal cancer, the incidence of postoperative complications remains high, reaching up to 35% (2). These complications are not only related to the surgery itself but are also influenced by the patient’s overall health and other non-surgical factors.

After potentially curative surgery for colorectal cancer, prognosis and treatment decisions are typically based on tumor pathology. However, an increasing body of research indicates that tumor staging is not the only factor determining patient outcomes. Body composition has increasingly been recognized as playing an important role in prognosis. Body composition parameters, such as obesity, are strongly linked to the prognosis of colorectal cancer. Epidemiological studies have shown that obesity is a significant risk factor for colorectal cancer (3). Furthermore, obesity is considered a risk factor for postoperative complications in various abdominal surgeries (4). However, the impact of obesity on colorectal cancer prognosis remains controversial. Some studies report worse outcomes in both obese (5, 6) and underweight patients (7), while others have not observed such associations (8).

Visceral obesity (VO) refers to the excessive accumulation of intra-abdominal adipose tissue and is generally considered a more accurate indicator of obesity than body mass index (BMI) (9, 10). Due to its central role in metabolism and its potential impact on postoperative recovery, accurately measuring visceral fat may have significant clinical value in the preoperative risk assessment of colorectal cancer patients. Compared to traditional BMI, computed tomography (CT) provides a more precise and reproducible method for measuring visceral fat area (VFA) (11). In the preoperative evaluation of colorectal cancer patients, abdominal CT imaging is routinely used not only to screen for metastatic disease but also to assess visceral fat accumulation.

This study aims to conduct a systematic review and meta-analysis to explore the impact of VO, measured exclusively by CT imaging, on the prognosis of colorectal cancer patients. Specifically, the study will evaluate the predictive value of visceral obesity for both short-term outcomes, such as postoperative complications, and long-term outcomes, including overall survival (OS) and disease-free survival (DFS), following elective colorectal cancer surgery.

## 2 Materials and methods

### 2.1 Search strategy

The search was conducted until October 2024, and primary studies were retrieved from databases such as Embase, PubMed, and Web of Science. “Colorectal cancer,” “visceral obesity,” and “visceral fat area” were among the search terms. The comprehensive search approach is available in **Supplementary Appendix 1**.

### 2.2 Inclusion and exclusion criteria

The following requirements had to be fulfilled for a study to be included: (1) all patients had been diagnosed with colorectal cancer and treated surgically; (2) patients had a CT scan prior to surgery, and VFA was measured; (3) the article includes intraoperative information or short-term or long-term postoperative outcomes. The study with more recent data collection or more extensive data was preferred among several papers written by the same researcher from a certain university.

Studies that satisfied any of the following requirements were disqualified: (1) did not employ CT scans to evaluate VFA for diagnosing VO; (2) did not compare patients with and without VO; (3) was not published in English; and (4) was a conference abstract, case report, review article, or letter.

### 2.3 Data extraction

Detailed information, including the names of the authors, study duration, year of publication, type of surgery performed, sample size, mean age of participants, gender distribution, study location, diagnostic criteria, length of hospital stay, operative time, intraoperative blood loss, number of lymph nodes retrieved, conversion rates, reoperation rates, postoperative readmission and mortality rates, overall and severe complication rates, the incidence of specific complications, and OS and DFS outcomes, was included in the compiled data.

### 2.4 Study quality

Using the Newcastle-Ottawa Scale (NOS), we assessed the calibre of the research that were part of our review. The maximum score on this scale is nine points. Research is considered methodologically sound if it receives a score of six or higher.

### 2.5 Statistical analysis

RevMan 5.4 and Stata 12.0 software were used for the data analysis. Weighted mean differences (WMD) were applied to continuous variables, odds ratios (OR) to binary variables, and hazard ratios (HR) to survival data. Heterogeneity between studies was evaluated using the I^2^ statistic. For I^2^ values of 50% or less, a fixed-effects model was used; for I^2^ values more than 50%, a random-effects model was used. To evaluate publication bias, funnel plots were employed in conjunction with Begg’s and Egger’s tests. Statistical significance was defined as a P-value of less than 0.05.

## 3 Results

### 3.1 Selected articles

**Figure 1:**
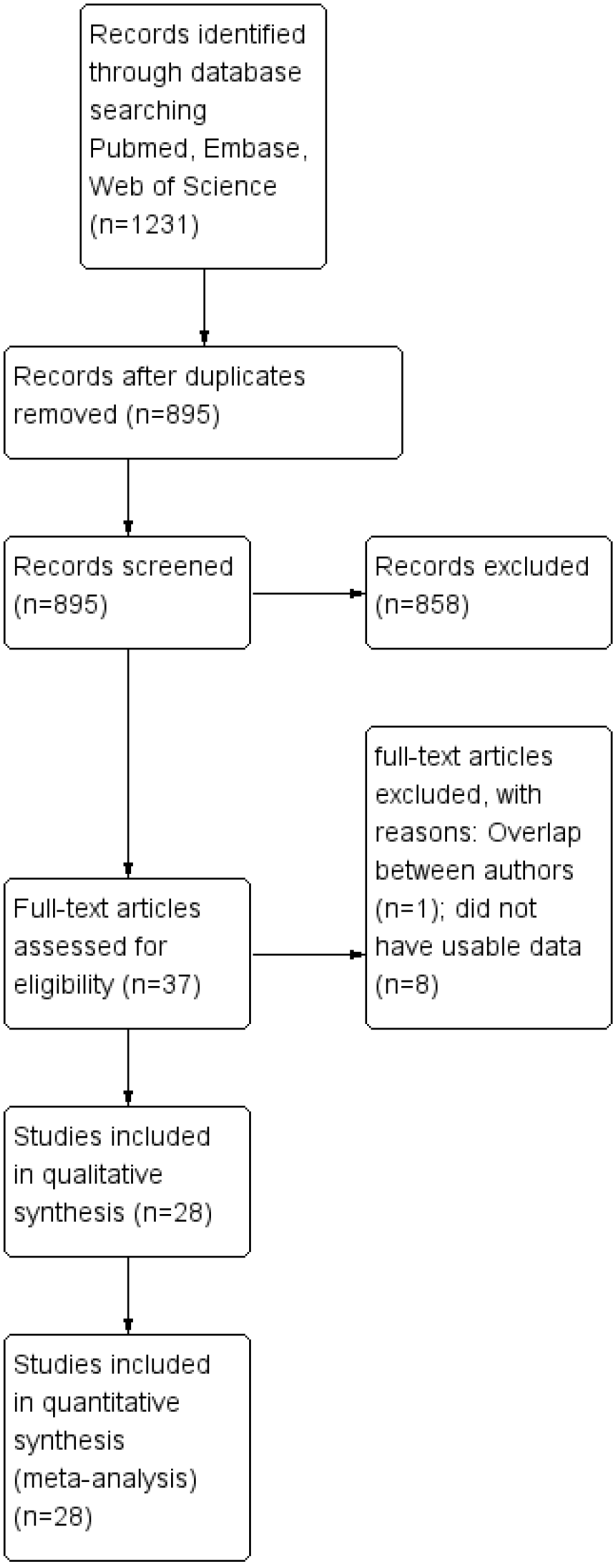
Flowchart of the search strategy.

1231 publications were selected during the first search phase. 28 (12–39) researches were eventually included in the meta-analysis after these publications were reviewed (**Figure 1**).

### 3.2 Study characteristics and study quality

The characteristics of the studies that were part of the analysis are compiled in **Table 1**. From 2005 to 2024, these studies were carried out in Japan, Korea, China, the Netherlands, the United Kingdom, the United States, Scotland, Canada, Italy, Chile, and Denmark. 5,756 individuals with VO and 5,373 patients without VO were among the 11,129 patients who had colorectal cancer resected. All of the publications that were part of this investigation had NOS values between 6 and 9, which meant that the study quality was adequate.

**Table 1:** Characteristics of the incorporated investigations.

Study	Nation	Year	Time period	Definition of VO	Definition of non-VO	Disease	Type of surgery	Patients		Female/Male		Mean age		Ottawa Scores
								VO	Non-VO	VO	Non-VO	VO	Non-VO	
Ishii	Japan	2005	1993-2002	≥100 cm^2^	<100 cm^2^	Colorectal cancer	Laparoscopic	9	37	1/8	21/16	N/A	N/A	6
Tsujinaka	Japan	2008	2001-2007	≥130 cm^2^	<130 cm^2^	Sigmoid colon cancer	Laparoscopic	68	65	19/49	35/30	65.5 (41–88)	64 (31–84)	7
Kang	Korea	2012	2003-2009	≥130 cm^2^	<130 cm^2^	Rectal cancer	Laparoscopic	29	113	13/16	40/73	67.5 ± 9.6	60.9 ± 10.6	6
Yamamoto	Japan	2012	2000-2005	Men: ≥ 130 cm^2^ Women: ≥90 cm^2^	Men: < 130 cm^2^ Women: <90 cm^2^	Colorectal cancer	N/A	102	171	38/64	62/109	66.7 ± 9.6	66.3 ± 10.5	6
Watanabe	Japan	2014	2005-2010	≥100 cm^2^	<100 cm^2^	Colon cancer	Laparoscopic	144	194	37/107	122/72	66.2 (35–90)	64.8 (35–87)	6
Cakir	The Netherlands	2015	2006-2013	>100 cm^2^	≤100 cm^2^	Colon cancer	Open and laparoscopic	367	197	130/237	147/50	71 ± 10	68 ± 12	6
Chen B	China	2016	2011-2014	≥100 cm^2^	<100 cm^2^	Rectal cancer	Laparoscopic	192	130	N/A	N/A	N/A	N/A	6
Malietzis	UK	2016	2006-2011	Male: ≥163.8 cm^2^ Female: ≥80.1 cm^2^	Male: <163.8 cm^2^ Female: <80.1 cm^2^	Colorectal cancer	Open and laparoscopic	420	385	N/A	N/A	N/A	N/A	6
Shiomi	Japan	2016	2017-2018	≥130 cm^2^	<130 cm^2^	Rectal cancer	Robotic and laparoscopic	82	154	65/69	89/65	NA	NA	7
Yu	Korea	2016	2011-2013	Male: >130 cm^2^ Female: >90 cm^2^	Male: ≤130 cm^2^ Female: ≤90 cm^2^	Colorectal cancer	Open and laparoscopic	22	80	12/10	24/56	65.1 ± 2.7	60. ± 1.1	6
Ozoya	USA	2017	2006-2015	>100 cm^2^	≤100 cm^2^	Colon cancer	Open and laparoscopic	82	28	32/50	18/10	66.2 ± 10.9	64 ± 14.5	6
Chen WZ	China	2018	2014-2017	Male: >130 cm^2^ Female: >90 cm^2^	Male: ≤130 cm^2^ Female: ≤90 cm^2^	Colorectal cancer	Open and laparoscopic	191	185	87/104	61/124	N/A	N/A	7
Choi	Korea	2018	2009-2013	>100 cm^2^	≤100 cm^2^	Rectal cancer	Open and laparoscopic	97	91	30/67	41/50	62.7 ± 11.4	59.9 ± 10.8	8
Almasaudi	Scotland	2019	2008-2016	Male: >160 cm^2^ Female: >80 cm^2^	Male: ≤160 cm^2^ Female: ≤ 80 cm^2^	Colorectal cancer	N/A	543	198	246/297	85/113	N/A	N/A	6
Heus	The Netherlands	2019	2006-2013	>100 cm^2^	<100 cm^2^	Rectal cancer	Open and laparoscopic	272	134	80/192	73/61	69 ± 9	65 ± 12	6
Hopkins	Canada	2019	2007-2009	Male: >160 cm^2^ Female: >80 cm^2^	Male: ≤160 cm^2^ Female: ≤ 80 cm^2^	Colorectal cancer	N/A	494	474	142/352	237/237	N/A	N/A	6
Morimoto	Japan	2019	2010-2012	≥100 cm^2^	<100 cm^2^	Colorectal cancer	Open and laparoscopic	156	261	N/A	N/A	N/A	N/A	6
Zhai TS	China	2019	2015-2017	≥100 cm^2^	<100 cm^2^	Colon cancer	Open and laparoscopic	73	34	32/41	11/23	69.6 ± 9.1	65.8 ± 14.5	6
Han	Korea	2020	2005-2012	≥100 cm^2^	<100 cm^2^	Rectal cancer	N/A	670	714	173/497	323/391	N/A	N/A	6
Pedrazzani	Italy	2020	2012-2019	Male: ≥163.8 cm^2^ Female: ≥80.1 cm^2^	Male: <163.8 cm^2^ Female: <80.1 cm^2^	Colorectal cancer	Laparoscopic	173	88	76/97	37/51	69.6 ± 11	64.4 ± 12.3	8
Cárcamo	Chile	2021	2010-2015	Male: >160 cm^2^ Female: >80 cm^2^	Male: ≤160 cm^2^ Female: ≤ 80 cm^2^	Colorectal cancer	Open and laparoscopic	263	96	N/A	N/A	N/A	N/A	6
Frostberg	Denmark	2021	2010-2011	≥130 cm^2^	<130 cm^2^	Colorectal cancer	Open and laparoscopic	130	148	30/100	83/65	71.5	70	6
Dong	China	2022	2015-2020	Male: >130 cm^2^ Female: >90 cm^2^	Male: ≤130 cm^2^ Female: ≤90 cm^2^	Colorectal cancer	Open and laparoscopic	261	267	123/138	87/180	74.0 ± 6.0	74.1 ± 6.4	6
Fujimoto	Japan	2022	2018-2020	≥100 cm^2^	<100 cm^2^	Colorectal cancer	Laparoscopic	46	78	N/A	N/A	N/A	N/A	6
Gachabayov	USA	2023	N/A	>100 cm^2^	≤100 cm^2^	Rectal cancer	Robotic	106	394	N/A	N/A	57.9 ± 12	59.3 ± 12.3	6
Zhai W	China	2023	2015-2021	≥100 cm^2^	<100 cm^2^	Colorectal cancer	Open and laparoscopic	337	183	212/125	95/88	N/A	N/A	6
Zhou	China	2023	2013-2019	≥100 cm^2^	<100 cm^2^	Rectal cancer	N/A	306	318	105/201	140/178	N/A	N/A	6
Zhao	China	2024	2019-2023	≥100 cm^2^	<100 cm^2^	Rectal cancer	Robotic	121	156	54/67	74/82	N/A	N/A	6

N/A not available, VO visceral obesity, non-VO non- visceral obesity.

### 3.3 Length of hospital stay

**Figure 2:**
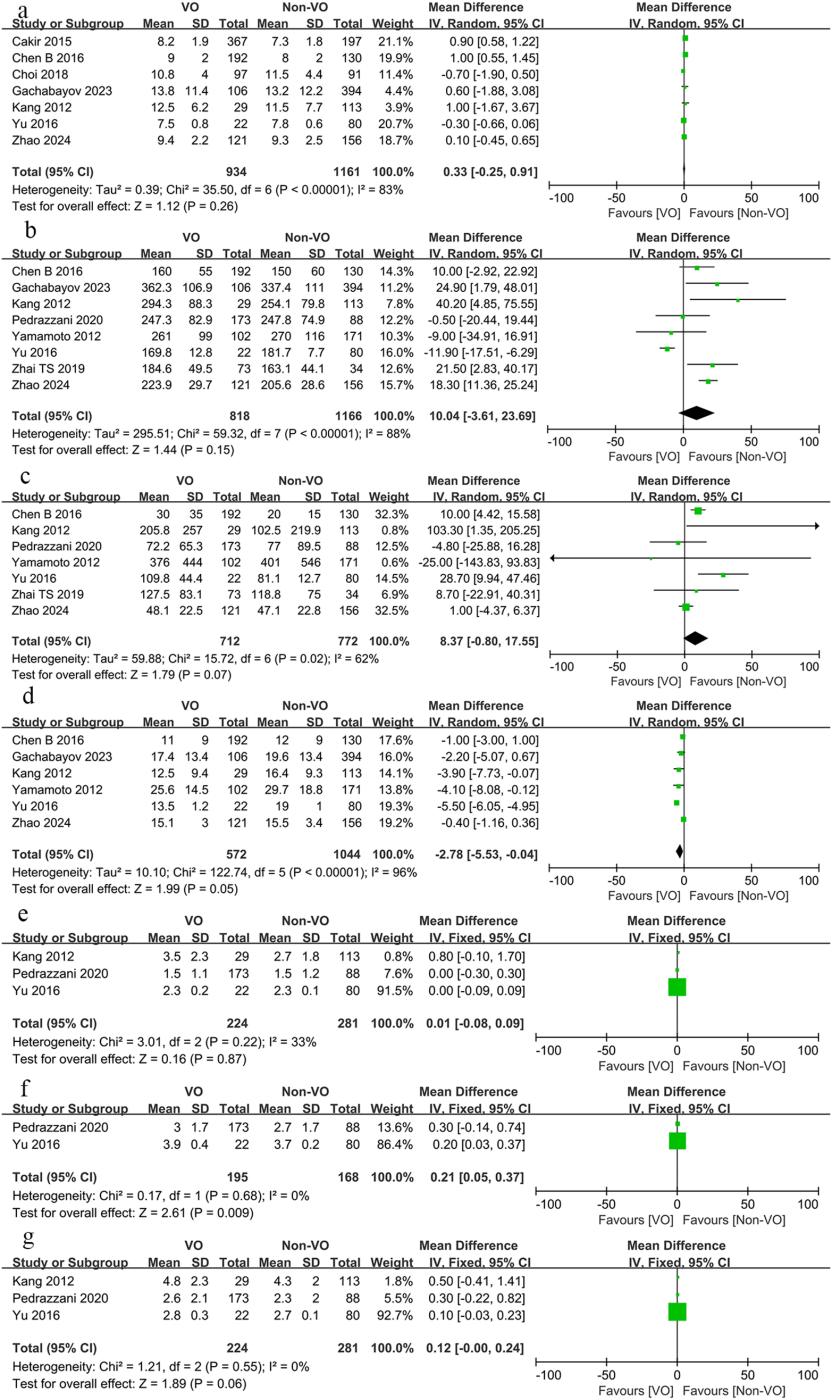
Forest plot describing the differences in **(a)** length of hospital stay, **(b)** operative time, **(c)** blood loss, **(d)** number of lymph nodes retrieved, **(e)** time to first flatus, **(f)** time to first bowel movement and **(g)** time to first food intake between VO and non-VO.

The average length of stay for the VO group ranged from 6 to 15.21 days, whereas for the non-VO group, it ranged from 6 to 13.2 days (**Table 2**). The length of hospital stay for the VO and non-VO groups did not differ significantly, as seen by **Figure 2a** (WMD, 0.33 days; 95% confidence interval [CI], -0.25 to 0.91; p = 0.26).

**Table 2:** Evaluation of intraoperative parameters and outcomes from each study.

Study	Length of hospital stay (days)		Operative time (min)		Blood loss (ml)		Conversion rate (%)		Lymph nodes retrieved	
	VO	Non-VO	VO	Non-VO	VO	Non-VO	VO	Non-VO	VO	Non-VO
Ishii	15 (6–55)	8 (5–74)	377 (276–550)	305 (184–590)	20 (10–945)	10 (10–965)	N/A	N/A	N/A	N/A
Tsujinaka	10.5 (5–31)	9 (5-29)	220 (125–410)	190 (115–325)	42.5 (10–530)	10 (10–890)	6 (8.8)	3(4.6)	N/A	N/A
Kang	12.5 ± 6.2	11.5 ± 7.7	294.3 ± 88.3	254.1 ± 79.8	205.8 ± 257.0	102.5 ± 219.9	5 (17.2)	6 (5.3)	12.5 ± 9.4	16.4 ± 9.3
Yamamoto	N/A	N/A	261 ± 99	270 ± 116	376 ± 444	401 ± 546	N/A	N/A	25.6 ± 14.5	29.7 ± 18.8
Watanabe	11.2 (6–320)	11.3 (6–53)	197 (86–576)	178 (55–319)	45.1 (0–400)	35.6 (0–1900)	N/A	N/A	23.6 (2–76)	30.8 (9–92)
Cakir	8.2 ± 1.9	7.3 ± 1.8	N/A	N/A	N/A	N/A	N/A	N/A	N/A	N/A
Chen B	9 ± 2	8 ± 2	160 ± 55	150± 60	30 ± 35	20 ± 15	N/A	N/A	11 ± 9	12 ± 9
Malietzis	6 (5–10)	7 (5–12)	N/A	N/A	N/A	N/A	N/A	N/A	N/A	N/A
Yu	7.5 ± 0.8	7.8 ± 0.6	169.8 ± 12.8	181.7 ± 7.7	109.8 ± 44.4	81.1 ± 12.7	0 (0)	2 (2.5)	19.0 ± 1.0	13.5 ± 1.2
Choi	10.8 ± 4.0	11.5 ± 4.4	N/A	N/A	N/A	N/A	N/A	N/A	N/A	N/A
Heus	11.3	9.4	166	149	431	310	6 (8)	3 (10)	N/A	N/A
Zhai TS	15.21 ± 7.59	12.29 ± 5.40	184.6 ± 49.5	163.1 ± 44.1	127.5 ± 83.1	118.8 ± 75.0	N/A	N/A	N/A	N/A
Pedrazzani	6 (2-68)	6 (3-53)	247.3 ± 82.9	247.8 ± 74.9	72.2 ± 65.3	77 ± 89.5	20 (11.6)	5 (5.7)	N/A	N/A
Dong	13 (7.5)	13 (6)	180 (80)	160 (85)	N/A	N/A	N/A	N/A	25 (6)	31 (5)
Gachabayov	13.8 ± 11.4	13.2 ± 12.2	362.3 ± 106.9	337.4 ± 111	N/A	N/A	N/A	N/A	17.4 ± 13.4	19.6 ± 13.4
Zhai W	13.00 (11.00, 16.00)	13.00 (11.00,15.00)	N/A	N/A	N/A	N/A	N/A	N/A	N/A	N/A
Zhao	9.4 ± 2.2	9.3 ± 2.5	223.9 ± 29.7	205.6 ± 28.6	48.1 ± 22.5	47.1 ± 22.8	3 (2.5)	3 (1.9)	15.1 ± 3.0	15.5 ± 3.4

N/A not available, VO visceral obesity, non-VO non- visceral obesity.

### 3.4 Operative time

The average operative time for the VO group ranged from 160 to 377 minutes, while for the non-VO group, it spanned from 149 to 337.4 minutes (**Table 2**). The operative duration for the VO and non-VO groups was not substantially different, as illustrated in **Figure 2b** (WMD, 0.15 minutes; 95% CI, -3.61 to 23.69; p = 0.15).

### 3.5 Blood loss

In the VO group, the average intraoperative blood loss was between 20 and 431 ml, whereas in the non-VO group, it was between 10 and 401 ml (**Table 2**). As **Figure 2c** illustrates, the difference was not substantial (WMD, 8.37 ml; 95% CI, -0.8 to 17.55; p = 0.07).

### 3.6 Number of lymph nodes retrieved

Compared to the non-VO group, fewer lymph nodes were recovered from the VO group (WMD, -2.87; 95% CI, -5.53 to -0.04; p = 0.05, **Figure 2d**).

### 3.7 Time to first flatus, bowel movement, and intake of food

There was no difference in the time to first flatus between the VO and non-VO groups (WMD, 0.01 days; 95% CI, -0.08 to 0.09; p = 0.87, **Figure 2e**). Although the time to first bowel movement was significantly shorter in the non-VO group compared to the VO group (WMD, 0.21 days; 95% CI, 0.05 to 0.37; p = 0.009, **Figure 2f**), the difference in the time to first food intake was not significant (WMD, 0.12 days; 95% CI, -0.00 to 0.24; p = 0.06, **Figure 2g**).

### 3.8 Conversion, reoperation, postoperative readmission and mortality rates

**Figure 3:**
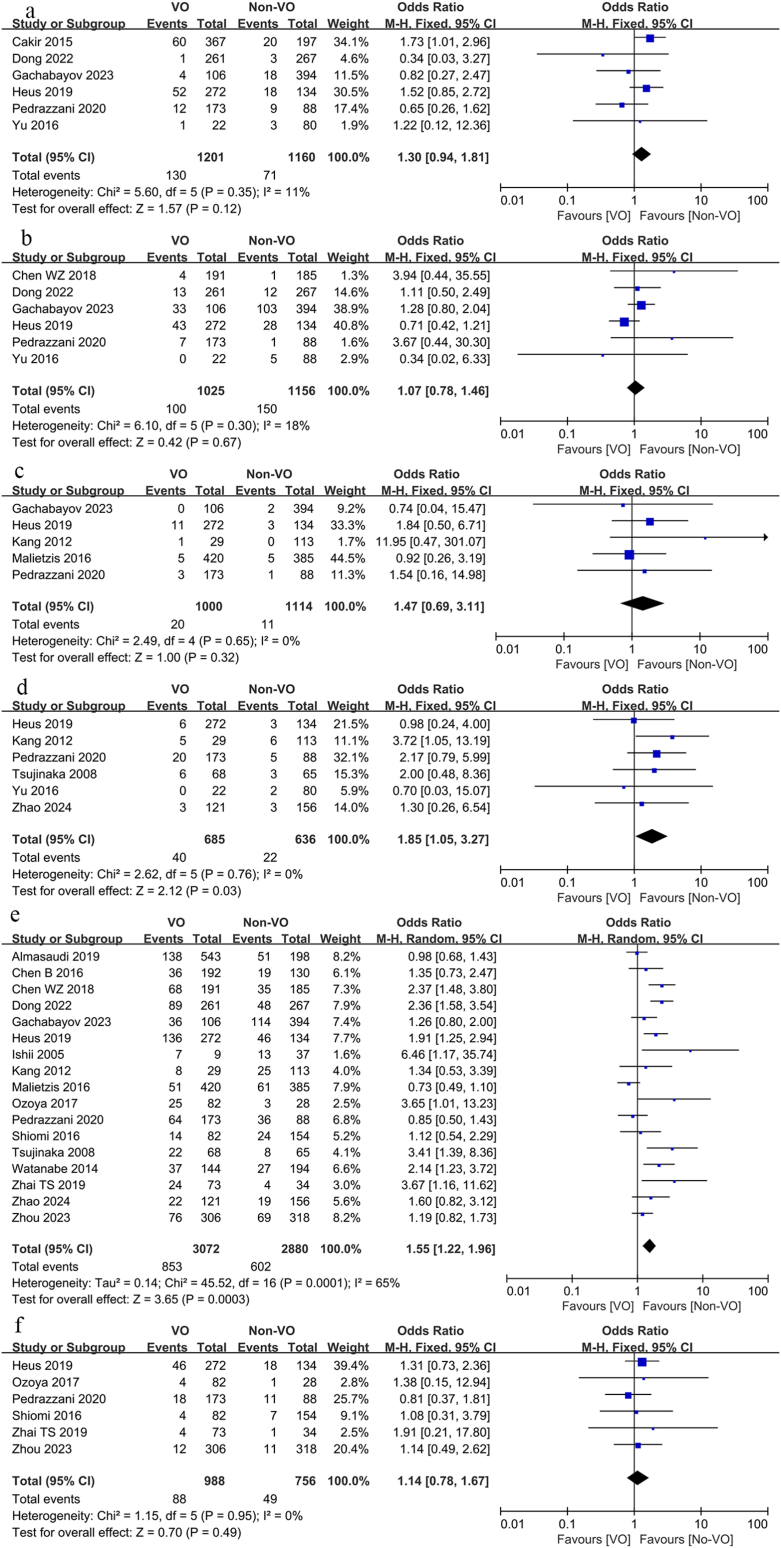
Forest plot describing the differences in **(a)** reoperation rate, **(b)** readmission rate, **(c)** mortality, **(d)** conversion rate, **(e)** total complications and **(f)** severe complications between VO and non-VO.

Reoperation (OR, 1.3; 95% CI, 0.94 to 1.81; p = 0.12; **Figure 3a**), postoperative readmission (OR, 1.07 95% CI, 0.78 to 1.46; p = 0.67; **Figure 3b**), and mortality rates (OR, 1.47; 95% CI, 0.16 to 2.53; p = 0.32; **Figure 3c**) did not significantly differ between the two groups, however the conversion rate in the VO group was significantly higher than that in the non-VO group (OR, 1.85; 95% CI, 1.05 to 3.27; p = 0.03; **Figure 3d**) (**Table 3**).

**Table 3:** Postoperative conditions and complications in each group.

Study	Postoperative mortality		Reoperation		Readmission		Overall Complications		Severe complications	
	VO	Non-VO	VO	Non-VO	VO	Non-VO	VO	Non-VO	VO	Non-VO
Ishii	N/A	N/A	N/A	N/A	N/A	N/A	7 (77.7)	13 (35.1)	N/A	N/A
Tsujinaka	N/A	N/A	N/A	N/A	N/A	N/A	22 (32.4)	8 (12)	N/A	N/A
Kang	1 (1)	0 (0)	N/A	N/A	N/A	N/A	8 (27.6)	25 (22.1)	N/A	N/A
Cakir	N/A	N/A	60 (16.3)	20 (10.2)	N/A	N/A	N/A	N/A	N/A	N/A
Chen B	N/A	N/A	N/A	N/A	N/A	N/A	36 (18.8)	19 (14.6)	N/A	N/A
Malietzis	5 (1.2)	5 (1.3)	N/A	N/A	N/A	N/A	51 (12.1)	61 (15.8)	N/A	N/A
Shiomi	N/A	N/A	N/A	N/A	N/A	N/A	14 (17.1)	24 (15.6)	4 (4.6)	7 (4.5)
Yu	N/A	N/A	1 (4.5)	3 (3.8)	0 (0)	5 (6.3)	N/A	N/A	N/A	N/A
Ozoya	N/A	N/A	N/A	N/A	N/A	N/A	25 (30.5)	3 (10.7)	4 (4.9)	1 (3.6)
Chen WZ	N/A	N/A	N/A	N/A	4 (2.1)	1 (0.5)	68 (35.6)	35 (18.9)	N/A	N/A
Almasaudi	N/A	N/A	N/A	N/A	N/A	N/A	138 (47)	51 (45)	N/A	N/A
Heus	11	3	52 (19)	18 (14)	43 (16)	28 (21)	136 (50)	46 (34)	46 (17)	18 (13)
Zhai TS	N/A	N/A	N/A	N/A	N/A	N/A	24 (32.9)	4 (11.8)	4 (5.5)	1 (2.9)
Pedrazzani	3 (1.7)	1 (1.1)	12 (6.9)	9 (10.2)	7 (4)	1 (1.1)	64 (37)	36 (40.9)	18 (10.4)	11 (12.5)
Dong	N/A	N/A	1 (0.4)	3 (1.1)	13 (5.0)	12 (4.5)	89 (34.1)	48 (18)	N/A	N/A
Gachabayov	0 (0)	2 (0.5)	4 (3.8)	18 (4.6)	33 (31.1)	103 (26.1)	36 (33.9)	114 (28.9)	N/A	N/A
Zhou	N/A	N/A	N/A	N/A	N/A	N/A	76 (24.8)	69 (21.7)	12 (3.9)	11 (3.5)
Zhao	N/A	N/A	N/A	N/A	N/A	N/A	22 (18.2)	19 (11.5)	N/A	N/A

N/A not available, VO visceral obesity, non-VO non- visceral obesity.

### 3.9 Complications

The total complication rate in the VO group exceeded that of the non-VO group (OR, 1.55; 95% CI, 1.22 to 1.96; p = 0.0003; **Figure 3e**), although there was no significant difference in the occurrence of severe complications between the two groups (OR, 1.14; 95% CI, 0.78 to 1.67; p = 0.49; **Figure 3f**) (**Table 3**).

**Figure 4:**
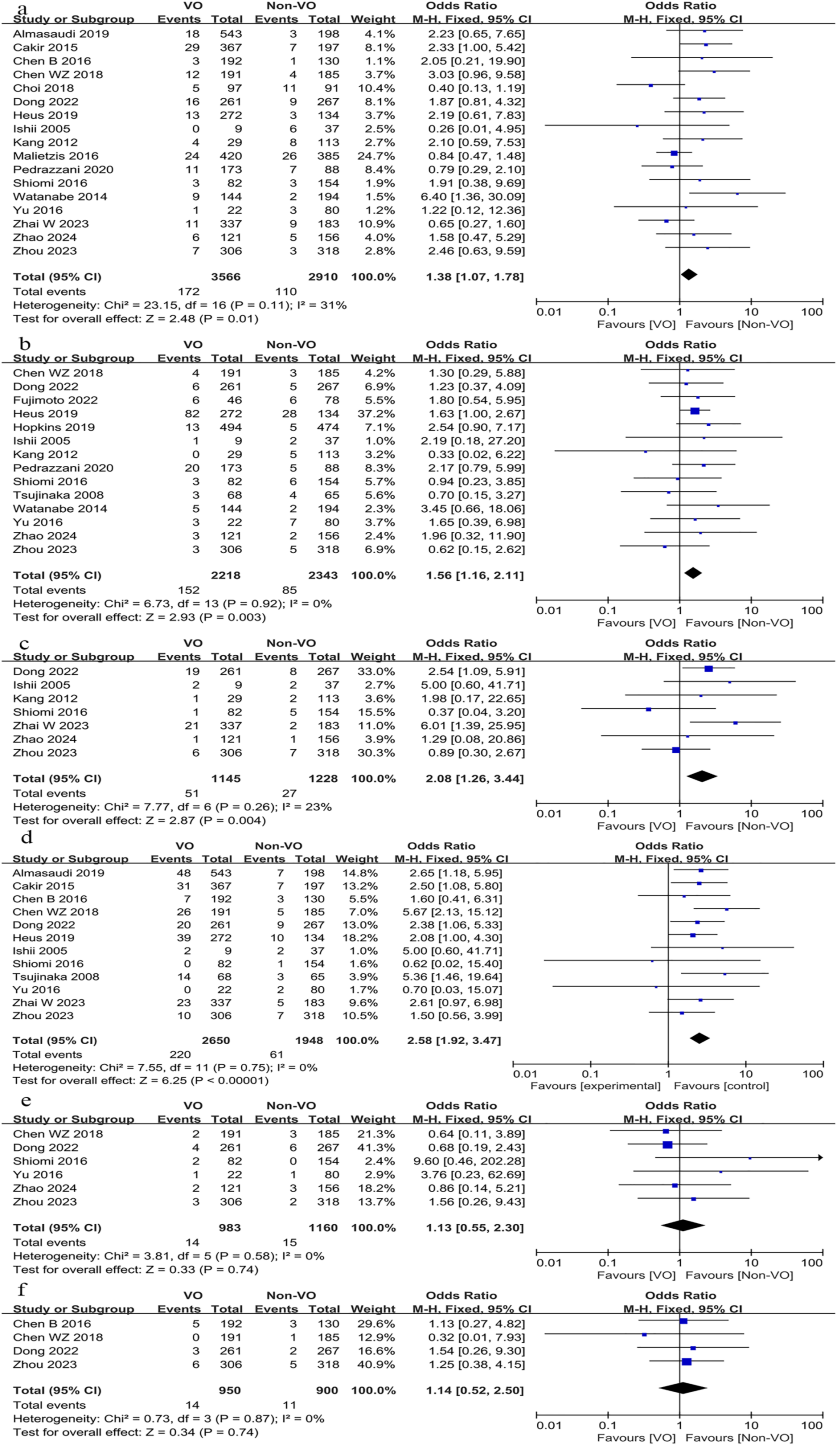
Forest plot describing the differences in **(a)** anastomotic leak, **(b)** intestinal obstruction, **(c)** intra-abdominal abscess, **(d)** wound infection, **(e)** bleeding and **(f)** gastrointestinal dysfunction between VO and non-VO.

**Figure 5:**
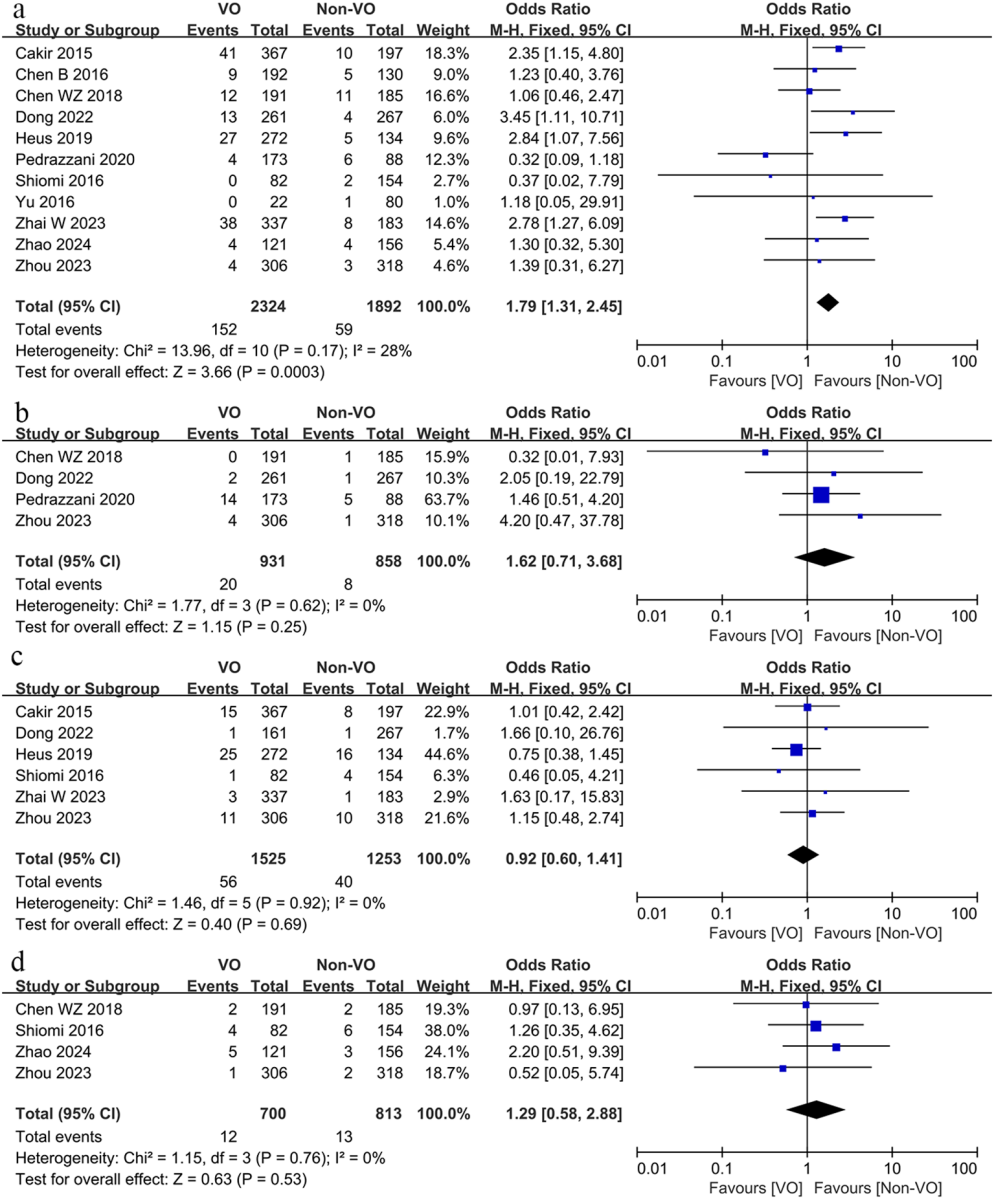
Forest plot describing the differences in **(a)** pulmonary problems, **(b)** cardiac issues, **(c)** urinary tract infections and **(d)** urine retention between VO and non-VO.

We subsequently compared complications associated with surgery, including anastomotic leak, hemorrhage, intestinal obstruction, intra-abdominal abscess, wound infection, and gastrointestinal dysfunction, alongside other complications such as pulmonary issues, cardiac complications, urinary tract infections, and urinary retention. The findings indicated that the occurrence of anastomotic leak (OR, 1.38; 95% CI, 1.07 to 1.78; p = 0.01; **Figure 4a**), intestinal obstruction (OR, 1.56; 95% CI, 1.16 to 2.11; p = 0.0003; **Figure 4b**), intra-abdominal abscess (OR, 2.08; 95% CI, 1.26 to 3.44; p = 0.004, **Figure 4c**), and wound infection (OR, 2.57; 95% CI, 1.92 to 3.47; p < 0.00001; **Figure 4d**) was markedly elevated in the VO group relative to the non-VO group, whereas no statistically significant difference was observed between the two groups regarding bleeding (p = 0.74; **Figure 4e**) and gastrointestinal dysfunction (p = 0.74; **Figure 4f**). The occurrence of pulmonary problems (OR, 1.79; 95% CI, 1.31 to 2.45; p = 0.0003; **Figure 5a**) was elevated in the VO group, although no significant disparity was observed in the frequencies of cardiac issues (p = 0.25; **Figure 5b**), urinary tract infections (p = 0.69; **Figure 5c**), and urine retention (p = 0.53; **Figure 5d**) between the two groups.

### 3.10 Prognosis

**Figure 6:**
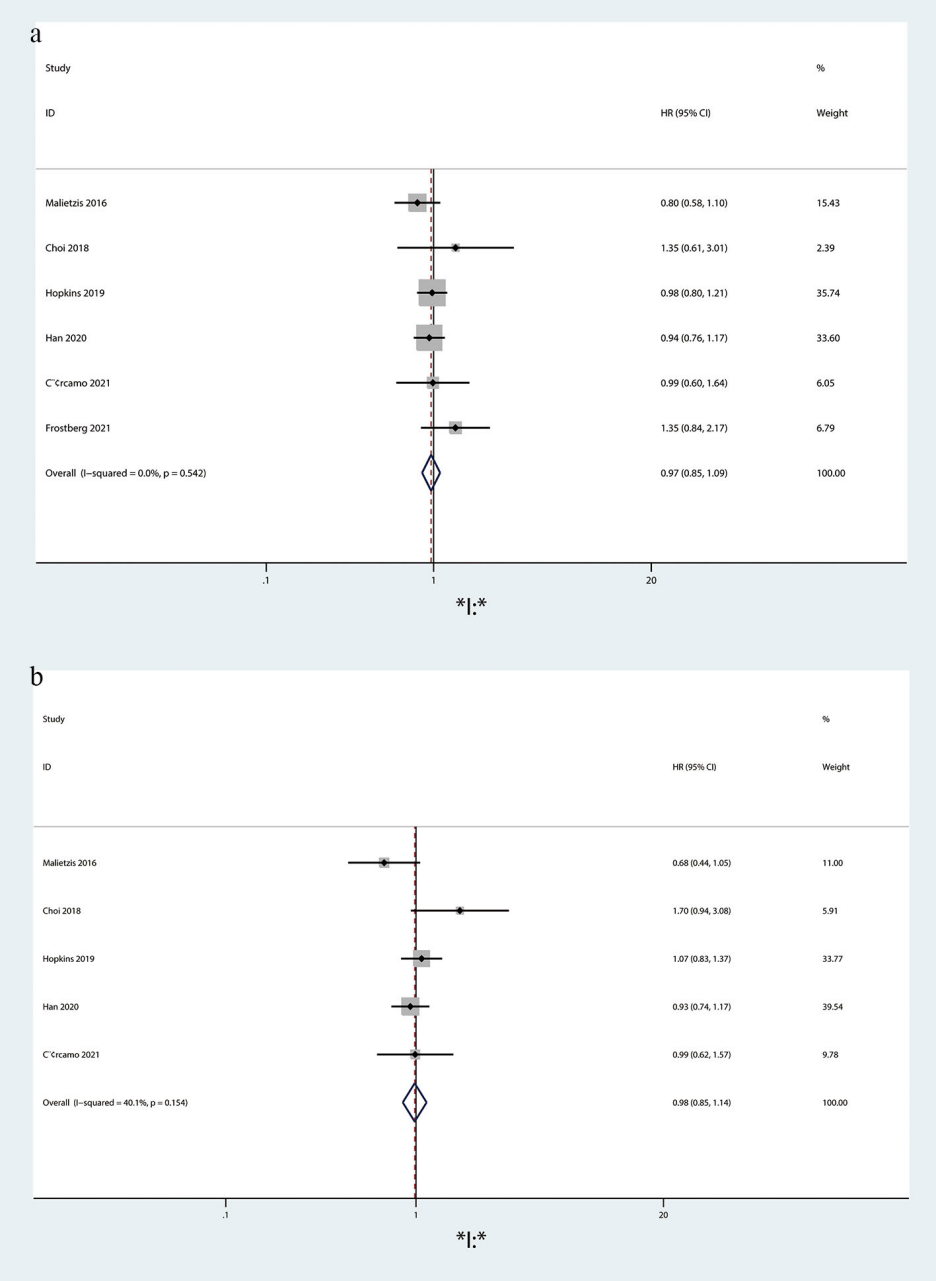
Forest plot describing the differences in **(a)** OS and **(b)** DFS between VO and non-VO.

OS (HR = 0.97, 95%CI = 0.85-1.09, p = 0.524; **Figure 6a**) and DFS (HR = 0.98, 95%CI = 0.85-1.14, p = 0.154; **Figure 6a**) did not differ statistically significantly between the VO and non-VO groups.

### 3.11 Publication bias

**Figure 7:**
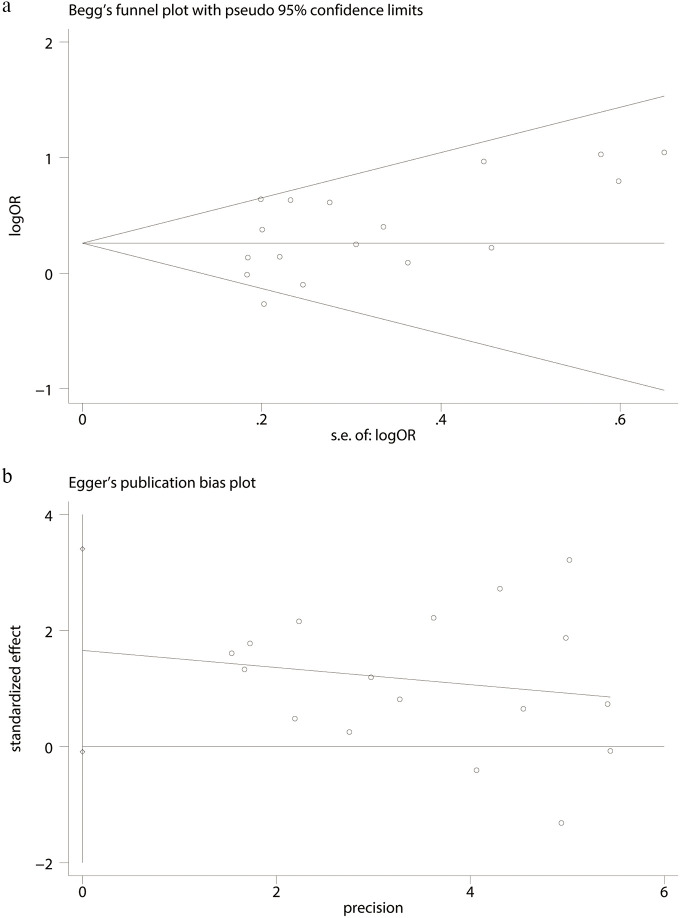
Test for publication bias: **(a)** Begg’s test and **(b)** Egger’s test.

Using the comprehensive complication data, Begg’s (p = 0.174; **Figure 7a**) and Egger’s tests (p = 0.061; **Figure 7b**) were performed, and no discernible publication bias was found.

## 4 Discussion

Obesity, being directly linked to several chronic illnesses and cancers, is a major global public health concern (40). Although BMI has long been the most commonly used metric for diagnosing obesity, it does not adequately reflect the distribution of fat in obese individuals. This is because obesity is characterized by an uneven distribution of fat tissue throughout the body, along with localized disruptions in fat and glucose metabolism (41). Obesity can be classified into two categories based on the location of fat accumulation: visceral obesity and peripheral obesity. The assessment of VFA through CT scanning is considered the gold standard for diagnosing visceral obesity, which is predominantly found in the abdominal region (42). Early studies have linked visceral fat to the incidence of CRC, the second most common cancer worldwide (43). However, the impact of VO on survival and surgical complications in colorectal cancer patients remains inconclusive (44, 45). Therefore, we conducted this meta-analysis to evaluate the effect of visceral obesity on postoperative outcomes in colorectal cancer.

We compared the data of colorectal cancer surgery patients with and without VO. We found that patients with VO experienced a longer recovery time for bowel movements, had fewer lymph nodes harvested, and had higher conversion rates. Additionally, the overall incidence of complications-including anastomotic leaks, intestinal obstruction, intra-abdominal abscesses, wound infections, and pulmonary complications-was significantly higher in patients with VO compared to those without. However, there was no difference in OS and DFS between the two groups.

The quantity of lymph nodes removed during tumor excision is a crucial predictor of oncological results in addition to demonstrating the surgeon’s ability. VFA influences the number of lymph nodes harvested, either because visceral obese patients have thicker mesenteries, which can make it difficult to see anatomical planes during surgery, making the surgical field more difficult and making it more difficult to dissect lymph nodes and identify blood vessels, which leads to fewer lymph nodes being harvested (14), or because fat tissue adheres to the mesentery, making it more difficult to identify lymph nodes in visceral obese patients (16). Despite the difficulty of dissecting lymph nodes, the operating time did not increase appreciably. The findings of meta-analyses that have already been published contradict this (46). We think that even with obese patients, the operational time can be reduced after a long learning period and overcoming the learning curve, even though the process is more complex.

In general, obesity, tumor size, and pelvic anatomical characteristics are the primary factors that contribute to the transition to open surgery (47, 48). Visceral obesity can further complicate the surgical field and operability, in addition to the challenges posed by limited anatomical features (49). This is specifically verified by our research.

Postoperative problems serve as critical indicators for evaluating short-term surgical outcomes, particularly in colorectal cancer surgery, where anastomotic leakage is a significant postoperative complication. Thickened mesentery, oversized omentum, and excessive visceral fat are frequently seen in patients with VO. VO is a significant predictor of surgical difficulties and patient outcomes because of these characteristics (50). Anastomotic leaking is more common in VO patients due to the increased technical difficulties of surgery caused by the growth in intra-abdominal fat. Furthermore, obesity raises intra-abdominal pressure for an extended period of time, which may hinder anastomotic site microcirculation and recovery (51). Metabolic problems are strongly associated with obesity, and the inflammatory state that these abnormalities cause may have a detrimental effect on anastomotic healing and tissue repair (52). Excess belly fat increases the risk of bowel blockage because it can impede or disrupt normal intestinal movement, which in turn increases the mechanical pressure on the intestines. Patients who are obese usually have thicker mesentery, which increases the risk of obstruction by causing insufficient blood flow to the intestines. According to research, inflammatory factors such IL-1α, IL-1β, and IL-1 receptor antagonists are more prevalent in human visceral fat tissue than subcutaneous fat tissue (53–55). Bowel obstruction is more likely to occur when the intestines are altered intraoperatively since this causes the release of these inflammatory substances. This also explains the delayed recovery of bowel function in the VO group, as evidenced by the prolonged time to first bowel movement after surgery. Furthermore, too much fat raises the danger of fat liquefaction and infection, which explains why wound and intra-abdominal infections are more common (56, 57).

Some negative outcomes can be improved with thorough preoperative evaluation and therapy. According to studies, visceral obesity can be successfully decreased by pharmaceutical therapies, dietary changes, exercise, and behavioral counselling (58). In addition to greatly reducing the risk of problems from surgery, preoperative reduction of visceral fat may also be taken into consideration for patients who have pneumonia or other pulmonary risk factors, especially if they are unable to bear additional respiratory harm. However, it takes time to reduce visceral fat, which may have negative consequences including tumor growth. As a result, it is crucial to perform a comprehensive evaluation of the patient’s general health and carefully consider the risks and timing of the intervention.

There is ongoing discussion over the effect of VFA on the long-term prognosis following colorectal cancer surgery. According to some research, VFA is a significant predictor of DFS following surgery for colorectal cancer (59) and is regarded as a separate risk factor for OS in patients undergoing adjuvant chemotherapy for the disease (60). Contrary to the above findings, new research indicates that patients with greater VFA scores actually have longer OS (44). Notably, our study’s findings indicate that there were no appreciable variations in long-term outcomes (OS and DFS) between the VO and non-VO groups among patients who had colon cancer surgery. There are several factors that affect the association between visceral fat and the long-term prognosis of colorectal cancer, some of which are not well supported by clinical data. We think that this association may be significantly influenced by variables including hormone levels (61), patient gender (62), and tumor stage (9). Visceral fat’s endocrine effects may encourage tumor growth and recurrence in patients with early and intermediate-stage cancer, which would lower OS and DFS. On the other hand, since cancer patients frequently have a negative energy balance and a larger visceral fat content may be advantageous for survival, a higher visceral fat content typically suggests superior nutritional reserves for patients with advanced disease. The long-term prognosis of patients with colorectal cancer may also be impacted by the notable variations in steroid hormone levels (such as testosterone and estradiol) between women before and after menopause. These hormones are strongly linked to fat deposition. Nevertheless, there is currently little clinical evidence to validate these putative determinants, and the study’s data set is small. To evaluate the effect of these factors on the long-term prognosis of patients with colorectal cancer, more extensive prospective studies are therefore required.

Considering the potential impact of our findings on clinical practice for colorectal cancer patients, this study highlights the importance of distinguishing between visceral and non-visceral obesity, which may lead to more targeted and effective interventions. By identifying visceral obesity as a key metabolic risk factor in colorectal cancer patients, clinicians can prioritize interventions such as lifestyle modifications or pharmacotherapy, especially for those with higher visceral fat accumulation. Furthermore, our findings may assist in the early identification of high-risk colorectal cancer patients for obesity-related comorbidities such as diabetes, cardiovascular diseases, and fatty liver, enabling more timely interventions and improving long-term outcomes. Moreover, incorporating the measurement of visceral fat into routine screening for colorectal cancer patients can help healthcare providers better stratify patient risks and personalize treatment strategies. Overall, this study emphasizes the importance of personalized obesity management, which could significantly improve clinical decision-making, patient outcomes, and public health interventions for colorectal cancer patients.

Among the limitations of this study are the following: (1) the definition of visceral obesity differed among the included studies (some defined it as VFA ≥ 100 cm^2^, others as VFA ≥ 130 cm^2^, and some studies classified it based on gender), (2) the surgical approaches used in the included studies varied (open surgery, laparoscopic surgery, and robotic surgery were used, which is another source of high heterogeneity and potential bias), and (3) there may be publication bias because the studies only included patients who had completed preoperative CT scans, while those without CT scans were excluded.

## 5 Conclusions

In summary, patients with VO who have colorectal cancer and have surgery tend to have fewer lymph nodes removed, higher conversion rates to open surgery, and more postoperative problems. Nevertheless, OS nor DFS seem to be substantially impacted by these factors. Large-scale, prospective research from other nations and areas are still required to confirm these findings and investigate the underlying mechanisms, though, because of sample size limits and regional diversity.

## Data Availability

The original contributions presented in the study are included in the article/**Supplementary Material**. Further inquiries can be directed to the corresponding author.
