# Supplementary material for: Impact of visceral obesity on postoperative outcomes in colorectal cancer: a systematic review and meta-analysis
Source: Front Oncol. 2025 May 6;15:1538073. doi: 10.3389/fonc.2025.1538073 (PMC12088942; doi:10.3389/fonc.2025.1538073)
Supplement: Supplementary file 1 [file DataSheet1.docx]

Search strategy:

(“VO” OR “visceral obesity” OR “visceral fat area”) AND (“rectal cancer” OR “rectal neoplasm” OR “rectal carcinoma” OR “colon cancer” OR “colon carcinoma” OR “colon neoplasm” OR “colorectal cancer” OR “colorectal carcinoma” OR “colorectal neoplasm”)
